# Kinesio Taping^®^ of the metacarpophalangeal joints and its effect on pain and hand function in individuals with rheumatoid arthritis

**DOI:** 10.4102/sajp.v72i1.314

**Published:** 2016-10-31

**Authors:** Sarah Roberts, Serela Ramklass, Robin Joubert

**Affiliations:** 1 Private Practice, South Africa; 2 School of Clinical Medicine, University of KwaZulu-Natal, South Africa; 3 Department of Occupational Theraphy, University of Kwa-Zulu Natal, South Africa

## Abstract

**Background:**

In persons suffering from rheumatoid arthritis (RA), the metacarpophalangeal (MCP) joints are commonly affected, resulting in inflammation, pain, joint instability, diminished grip strength and difficulties with function. However, the effectiveness of Kinesio Taping^®^ of the MCP joints in alleviating the symptoms has not been established.

**Aim:**

To determine the effectiveness of bilateral Kinesio Taping^®^ of the MCP joints on pain, range of motion, grip strength and hand function in elderly individuals previously diagnosed with RA.

**Methods:**

A repeated measure, experimental design was used over a 7-week period with the Kinesio Taping group (*n* = 30) receiving bilateral space correction Kinesio Taping^®^ of the MCP joints with a joint protection (JP) group (*n* = 31) participating in JP workshops. The Kinesio Tape^®^ was worn for 3 days a week with four applications during the data collection process. For the control group, 2-hour JP educational–behavioural workshops were run weekly for 4 weeks. Weekly assessments were completed for grip strength, ulnar deviation and pain (VAS), and two pre-intervention assessments and one post-intervention assessment was completed for the Michigan Hands Outcomes Questionnaire (MHQ).

**Results:**

Kinesio Taping^®^ of the MCP joints showed a significant decrease in pain (*p* = 0.001) and improved range of motion (*p* = 0.001 bilaterally). JP was found to have a significant improvement in grip strength (*p* = 0.001 bilaterally) and in the work (*p* = 0.01) and activities of daily living (ADL) (*p* = 0.01) sections of the MHQ. No significant difference was found between groups after intervention in the majority of outcomes (*p* = 0.24) except for grip strength where a significant difference was found.

**Conclusion:**

Kinesio Taping^®^ of the MCP joints is an effective conservative intervention to improve pain and MCP ulnar deviation in individuals with RA.

Significance of the work: Kinesio Taping^®^ of the MCP joints of individuals with RA showed a significant decrease in pain (*p* = 0.00) and improved range of motion (*p* = 0.001 bilaterally). JP was found to significantly improve grip strength (*p* = 0.001 bilaterally) and in the work (*p* = 0.01) and ADL (*p* = 0.01) sections of the MHQ. No significant difference was found between groups after intervention in the majority of outcomes (*p* = 0.24) except for grip strength where a significant difference was found (*p* = 0.001).

## Introduction

Rheumatoid arthritis (RA) is a chronic systemic disease that affects the hands bilaterally, resulting in inflammation, pain, joint instability, diminished grip strength and difficulties with function. The metacarpal joint (MCP) and hand function are affected in 65% of people with RA (Goosens *et al*. 2000) owing to the nature of the patho-mechanics surrounding this joint. Various interventions have been found to alleviate the symptoms associated with RA. However, the efficacy of assistive devices, whilst widely prescribed, has not been extensively researched (Beasley 2011; Steuljens *et al*. 2003; Tuntland *et al*. 2009). Splinting is found to alleviate pain, whilst joint protection (JP) displays strong evidence in assisting with function (Steultjens *et al*. 2003). Pain, stiffness, function and grip strength improve through the use of JP workshops (Alderson *et al*. 1999), specifically *educational–behavioural* JP programmes (Dures 2012; Hammond 2013; Iversen, Hammond & Betteridge 2010).

Hand function has also been shown to improve with hand exercises that are aimed at improving strength, endurance and mobility (Lamb *et al*. 2015). There is little evidence to support the effectiveness of other therapeutic modalities, in particular thermal modalities (Beasley 2012). Acupuncture-like transcutaneous electrical nerve stimulation has been found to assist with pain management (Brosseau *et al*. 2003). The effect of Kinesio Taping^®^ on the symptoms of RA is under-researched. Kinesio Taping^®^ has been found to be effective in the relief of short-term pain, improved range of movement and muscle strength (Anandkumar, Sudarshan & Nagpal 2014; Djordjevic *et al*. 2012). Effectiveness of Kinesio Taping^®^ in RA is often anecdotal in nature. Therapists who make use of Kinesio Taping^®^ for the symptoms of RA in the hand tend to base their treatment on their own experience and knowledge of the application of the tape for other conditions. Kinesio Taping^®^ is a treatment modality that has gained popularity amongst various rehabilitation disciplines. It is reportedly a convenient modality to use as it is fairly simple to teach a person the application of the taping (Coopee 2011). In addition, previous research has indicated that no adverse effects of the tape have been noted, making it an easily tolerated intervention (Beasley 2011)

Kinesio Taping^®^ is a proprioceptive tape composed of polymer elastic strands and cotton fibres (Kase, Wallis & Kase 2003). The properties of the tape, that is, Kinesio^®^ Tex Gold^TM^ (hereafter named the tape), are as follows: the thickness of the tape is set to mimic the thickness of the epidermis of the skin to prevent unnecessary sensory stimuli through the weight of the tape. Thus, the person wearing the tape will not be aware of it after 10 minutes of wear. The cotton fibres in the tape allow for the evaporation of body moisture and rapid drying. In addition, it is coated in paraffin so as to limit the amount of water absorption and is designed in a wave pattern which further allows the skin to breathe and also allows for evaporation of body moisture. The polymer elastic fibres run longitudinally with a stretch of up to 55%–60% of the resting length, mimicking the elastic capabilities of the skin. There is no horizontal stretch. These elastic fibres last for approximately 3–5 days. The tape is latex-free and the adhesive is 100% acrylic, thereby ensuring less allergenic properties (Coopee 2011; Kase *et al*. 2003). Application of the tape onto the skin provides a low-intensity stimulus which is postulated to have a reduction in pain.

In addition, Golgi tendon organs/receptors are stimulated through the application of the tape. Depending on the direction of pressure away or towards these receptors, inhibition or facilitation of the muscle can be stimulated. With regard to taping to facilitate muscle activity, research indicates that increased muscle activity is obtained between 24 and 72 hours whilst a decrease in muscle activity is noted on the fourth day of wearing the tape (Slupick *et al*. 2007). With regard to improving grip strength in hands, discrepancies have been found as to the effect of Kinesio Taping^®^ in healthy participants. Fratocchi *et al*. (2011) found that Kinesio Taping^®^ over the biceps brachii improves eccentric elbow torque. Kuo and Huang (2013) found that applying Kinesio Tape^®^ in different directions according to the muscle’s origins and insertions plays a role in the effect of Kinesio Taping^®^ on muscle strength in the wrist and fingers. Lee, Woo and Lee (2010) found that Kinesio Taping^®^ of the flexor muscles in the forearm increase grip strength, whilst Merino-Marban, Mayorga-Vega and Fernandez-Rodríguez (2012) found no changes in grip strength when the flexor muscles are taped. However, Mohammadi *et al*. (2014) found that grip strength increases following Kinesio Taping^®^ of the extensor muscles of the forearm specifically half an hour after taping in men and one-and-a-half hours following taping in women. When taping around joints, both pain receptors and proprioceptors are stimulated in order to increase support to the ligament and muscular structures working on that joint, thereby aligning the joint, reducing the pain and improving the range of motion. Kinesio Taping^®^ is also used to assist in the alignment of the fibres in fascia in order to increase mobility of tissues and reduce joint stiffness. Kinesio Taping^®^ is used through a different technique to lift the skin, thereby creating wrinkles in the skin and channels in the underlying tissue. These channels assist with lymphatic drainage as areas of differing pressure are created to move the lymph. In addition, the pressure of the tape does not block the lymphatic system and, besides creating channels, it stimulates the lymphatic system through the movement of the tape on the skin as it moves within its elastic properties. Deeper lymphatic drainage can be stimulated through facilitation of muscle contraction through use of the tape. Kinesio Taping^®^ is known to improve the range of motion in the following conditions: shoulder impingement, post knee surgery, patellofemoral pain syndrome, musculoskeletal pain in the lower back, whiplash, mechanical neck pain and sub-acute lateral ankle sprain (Djordjevic *et al*. 2012).

The aim of this study was to compare the effectiveness between patients receiving bilateral Kinesio Taping^®^ of the MCP joints and those receiving advice on JP upon symptoms of pain, range of motion, grip strength and hand function in elderly individuals previously diagnosed with RA, aged 50–80 years of age. This study was thus conducted to determine whether Kinesio Taping^®^ is effective in alleviating MCP joint symptoms in people with RA in order to understand whether it can be used as one of the conservative methods in the treatment of RA.

## Methodology

### Research setting

A repeated measure, experimental design was used over a 7-week period. The comparison between the pre-test and post-test measures gave an indication of the effect of the application of Kinesio Taping^®^, especially with regard to the immediate pre-intervention and immediate post-intervention measures over a short study period (Bless, Higson-Smith & Kagee 2007). The Kinesio Taping^®^ (KT group) group received four applications of Kinesio Taping^®^ and the JP workshop group (JP group) received four JP workshops. Three pre-test measures and four post-test measures were conducted on a weekly basis, measuring ulnar deviation, grip strength and pain. In addition, participants completed a subjective measure on weeks 1, 3 and 7. The JP group received an intervention that applied the JP principles. The KT group was not part of the JP workshops because any statistical difference found with the Kinesio Taping^®^ and JP workshops completed together compared with JP workshops alone would not have indicated the effectiveness of Kinesio Taping^®^ but would rather have been conclusive of the combination of therapeutic interventions.

### Study design and sample

Using convenience sampling, the study sample was drawn from seven retirement homes in the Pietermaritzburg and Midlands areas of KwaZulu-Natal (codenamed facility A–G). By including only retirement facilities within Howick and Pietermaritzburg, it ensured participants were exposed to similar weather environments. This was important to allow for consistency as studies have shown that the weather may affect pain in certain individuals who have been diagnosed with RA (Smedslund & Hagen 2011).

Using inclusion and exclusion criteria, participants were voluntarily recruited from each retirement facility. Inclusion criteria included people previously diagnosed with RA of the hand and who were currently experiencing pain in their hands owing to the RA whilst exclusion criteria included all contra-indications to the use of Kinesio Taping^®^, that is, malignancy, cellulitis, open wounds on the hands, infections, deep vein thrombosis, kidney disease, congestive heart failure conditions, previous surgery to the hand, deformities of the hand, other conditions affecting the hands, participation in other therapies or research related to the hand and difficulty completing the subjective questionnaires.

A total of 80 potential participants were recruited. Seven participants did not meet the inclusion criteria and nine individuals declined to participate. Therefore, a total of 64 participants were obtained for the sample. Retirement facility A was able to identify 34 participants (initially 32 with a further 2 being added later) and, therefore, the initial 32 participants formed the JP group being a convenience sample. The remaining two participants from this retirement facility as well as all participants from the other retirement facilities (B–G) (30) were included in the KT group, also as a convenience sample. Therefore, each group had a total of 32 participants. One participant withdrew from the KT group before the intervention began as she experienced a severe flare-up. Another participant withdrew from the same group following 4 weeks of data collection as she found the intervention too cumbersome. In the JP group, one participant withdrew because his spouse became unwell. Therefore, 30 participants in the KT group and 31 participants in the JP group completed the data collection. All participants in both groups completed a background information questionnaire prior to the beginning of the data collection process.

Apart from measuring increase or decrease in symptoms such as pain, range of motion and muscle strength, the effects of symptoms upon activities of daily living (ADL) were also measured.

#### Range of motion – MCP ulnar deviation

Bilateral MCP joint ulnar deviation was assessed dorsally using a 15 cm clear plastic goniometer with a 360 degree head that had 3 scales all calibrates for use with the International Standards of Measurement (ISOM) (Hitech Therapy 2013). A standard protocol and one tester completed all assessments in this study to assure reliability (Cambridge-Keeling 1995). Guidelines of the American Society for Surgery to the Hand (1990) for the measuring of MCP ulnar deviation were used. Three measures of each finger’s ulnar deviation at the MCP joint were completed, with the mean of the three being taken to ensure reliability of the data. Ulnar deviation of each of the MCP joints in both hands was assessed every week for 7 weeks.

#### Grip strength

Bilateral grip strength was measured using a calibrated Jamar Hydraulic Hand Dynamometer (Item # 08-1028950). This measures the isometric grip force on a dual scale from 0 to 90 kg or 0 to 200 pounds. The peak hold needle automatically retains a peak reading until the gauge is reset. The handle on the dynamometer can be adjusted to five different settings ranging incrementally in 13 mm increases from 35 to 87 mm (Hitech Therapy 2013). When it is calibrated correctly and used in situations that can be repeated, it has been shown to be a repeatable, sensitive test and an accurate instrument of the force of a person’s grip (Bell-Krotoski, Breger-Lee & Beach 1995). Specifically with regard to the elderly, the Jamar Dynamometer has been found to have test–retest reliability over a period of 12 weeks (intra-class correlation coefficients 0.954 and 0.912 for right and left hands, respectively) (Bohannon & Schaubert 2005).

Studies have shown that one pain-free grip strength test–retest measurement in RA is reliable when compared with the mean of three measurements with the intra-class correlation coefficient being ≥ 0.91 for both the one grip measurement and mean of three grip measurements (Kennedy, Jerosch-Herold & Hickson 2010). Therefore, only one test of pain-free grip strength (at the second spacing) was completed in order to limit the amount of pain experienced by the participant, and each participant’s assessment was completed at approximately the same time of day throughout the measures.

The standard testing position was used which necessitated that the participant was seated, shoulder adducted, forearm in neutral and elbow flexed to 90 degrees and the wrist position self-selected by the participant (Seftchick *et al*. 2011).

#### The Michigan hand outcomes questionnaire

The MHQ was used to assess the overall outcomes related to RA (Chung *et al*. 1998, 1999). The MHQ is a self-reported measure and has been found to be a responsive, reliable and valid measure in individuals with RA (Adams *et al*. 2010). The MHQ measures bilateral hand function, ADL, pain, work performance, aesthetics and patient satisfaction with hand function. An overall MHQ score is computed using all of the sections scored. Scores are reported as a percentage, with a higher score denoting better performance in that area. Participants self-completed the MHQ on weeks 1, 3 and 7.

#### Visual analogue scale

In addition to the MHQ, a visual analogue scale (VAS) of 100 mm was used to assess pain where 0 represented no pain and 10 represented the worst pain the participant has experienced (Fedorczyk 2011). The VAS has been shown to be reliable in terms of test–retest reliability and validity has been established (MacDermid 2011). Participants were requested to indicate on the VAS the average pain that they had experienced in their hands over the past week. In addition, each participant was asked to indicate, on a diagram of a hand, where they had experienced the pain in their hands. Participants completed the VAS every week.

### Data collection

A pilot study was conducted by the first author in order to test the use of the screening tool and assessment instruments. Six participants were recruited for the pilot study. Participants were identified through purposive sampling and all met the inclusion criteria. Following the pilot study, minor formatting changes were made to the informed consent document, screening questionnaire and the MHQ.

Three pre-test measures and four post-test measures of MHQ, ulnar deviation, grip strength and VAS were completed for each participant in the KT and JP groups. These measures were completed the same day, every week at approximately the same time. The pre-test measures were completed over a period of 3 weeks prior to any intervention.

**FIGURE 1:**
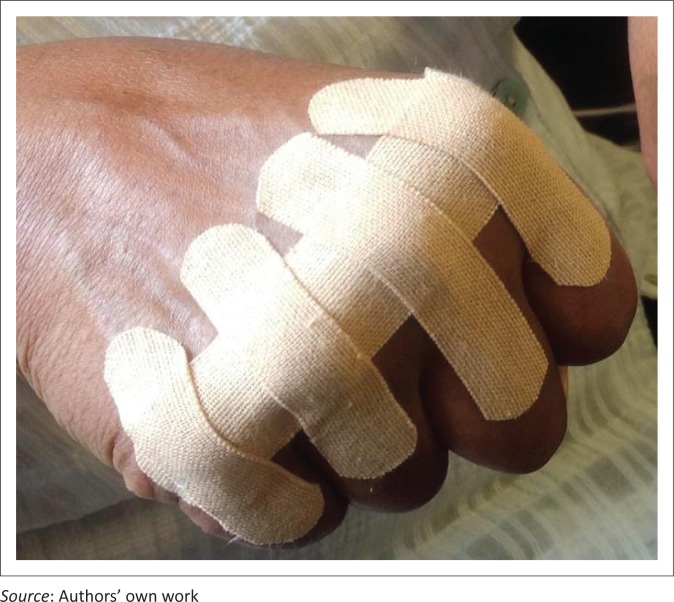
Application of Kinesio Taping^®^.

Kinesio Taping^®^ using the tape was applied for the KT group, beginning in week 3 of the data collection period. Each participant had the tape applied four times over a period of 4 weeks, and the tape was worn for 3 days on each occasion. The tape was applied as indicated in Figure 1, that is, over the MCP joints bilaterally owing to the higher percentage of involvement of the MCP joint in RA. It was isolated to the MCP joint to exclude confounding variables such as the impact of the tape on the extrinsic muscles of the forearm or RA pathology in the wrist were the tape to cross the wrist joint. An I-strip over all of the MCP joints was first applied with individual I-strips over each joint being placed at 90 degrees to the first tape. This second strip is postulated to provide further feedback through the tactile system in order to increase motor control of the joint (Simoneau *et al*. cited in Garcıa-Muro, Rodrıguez-Fernandez & Herrero-de-Lucas 2010). Bilateral taping of the MCP joints was completed.

The taping was completed using the procedure of a space correction application as this assists with pain reduction. Space correction is created through Kinesio Taping^®^ in order to lift the skin and thereby reduce the pressure from areas of pain, inflammation or oedema, either decreasing the stimulation to the receptors in order to alleviate pain and/or create channels for increased circulation. It is also postulated that the mechanoreceptors are stimulated, thereby initiating the gate control theory of pain. Space correction is completed through placing the soft tissue on stretch – in this instance, having the person fully flex his or her MCP joints, laying down the middle section of the tape with less than 50% stretch and lastly fixing the two tails (anchors) of the tape.

**FIGURE 2:**
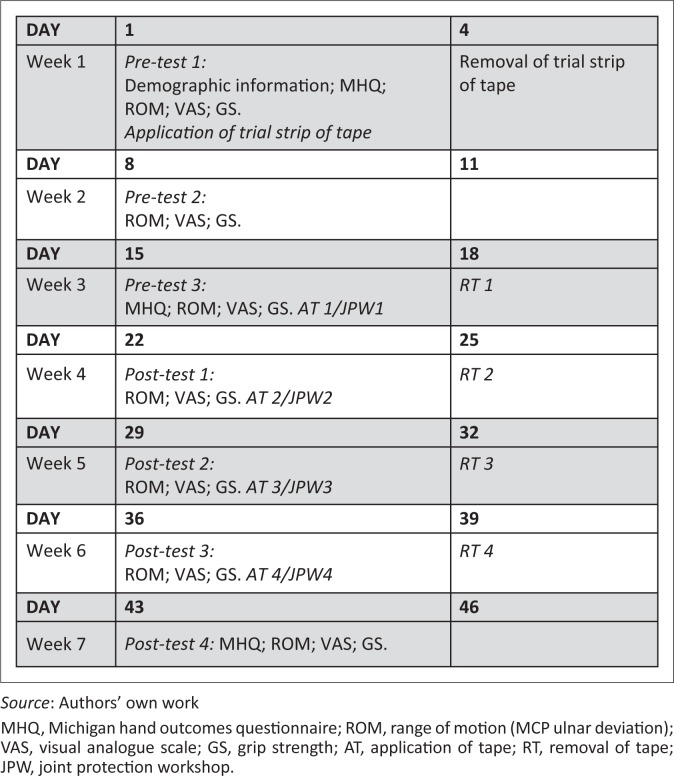
Representation of the procedures for the Kinesio Taping^®^ group and JP workshop group.

On each occasion, the tape was removed after 3 days of wearing as the efficacy of the tape on muscle facilitation is apparent during the first 3 days of application but wanes from the 4th day onwards (Slupick *et al*. 2007). As the effects of the tape have been shown to continue for a further 48 hours after removal, it was not reapplied for a further 4 days (Slupick *et al*. 2007). This allowed the skin of the participants to rest, especially as some of the participants had fragile or thin skin because of advanced age. No adverse reactions to the tape were reported by any of the participants. The data collection procedure for the assessment and intervention of the Kinesio Taping^®^ group is outlined in Figure 2.

JP workshops of 2 hours’ duration were conducted with the JP group by the first author for 4 weeks after completion of the three pre-test measures. The workshops included information sharing on RA and JP principles, creating self-awareness of RA symptoms and JP behaviours, demonstration and practice of JP skills using assistive devices and weekly goal setting in terms of JP principles and skills. Participants were given an information sheet on JP at each session.

JP sessions were completed as guided by Hammond and Lincoln (1999). The first session included theoretical information on RA and JP principles and energy conservation whilst later sessions included practical and theoretical information on assistive devices, splinting, diet, exercise, rest, relaxation, paraffin wax and pharmaceutical interventions. Each week, the participants were given a homework task to identify an activity for application of JP principles they had learnt during the session and practice of this task at home. Feedback on these tasks was discussed in the sessions that followed.

The procedure for the JP workshop group’s assessment and intervention (JP workshops) is indicated in Figure 2.

### Data analysis

Data were analysed using SPSS version 22. Calculation of the mean, standard deviation, frequencies and percentages was used to summarise data for both the experimental and control groups. Within each group, the paired *t*-test was used to compare symptoms of arthritis before and after each intervention (Table 1). The independent samples *t*-test was used to compare the characteristics of arthritis between the experimental and the control groups. The level of significance was set at *p* = 0.05. Cohen’s effect size (standardised difference in means) was calculated in order to determine the degree to which the difference between the two interventions was clinically significant (McGough & Faraone 2009). Standardised differences in means were quantitatively recorded. Repeated measure analysis of variance (ANOVA) was not utilised as the power of the sample size was inadequate.

**TABLE 1:** Paired *t*-test for changes over time for pain, MCP ulnar deviation, grip strength and overall MHQ score for the experimental group and control groups.

Variable	Experimental group			Control group		
	Mean	Standard deviation	*p*	Mean	Standard deviation	*p*
Pain (VAS)	2.7	2.39	0.01*	0.35	1.52	0.20
Overall pain score (MHQ)	−6.85	13.81	0.01*	2.55	14.30	0.33
Ulnar deviation – right hand	3.44	3.90	0.001*	0.63	2.16	0.12
Ulnar deviation – left hand	3.62	3.10	0.001*	0.44	2.10	0.25
Grip strength – right hand	0.16	2.75	0.76	-2.30	2.50	0.00*
Grip strength – left hand	0.48	1.66	0.14	-2.52	2.11	0.00*
Overall MHQ score	2.62	10.34	0.18	1.20	5.59	0.24

*Source*: Authors’ own work

MHQ, Michigan hand outcomes questionnaire; VAS, visual analogue scale;

* statistical significance.

## Ethical considerations

Ethical clearance for the study was granted by University of KwaZulu-Natal’s Biomedical Research Ethics Committee (BREC), certificate no. BFC183/12.

## Results

Sixty-three participants were screened for the study, with 32 participants in the KT group and 31 participants in the JP group and all meeting the inclusion criteria. Two participants withdrew from the KT group. The final profile of both groups is represented in Table 2.

**TABLE 2:** Profile of participants in experimental and control groups.

Characteristics	Experimental group	Control group
	*N* = 30	*N* = 31
Gender		
Male	5 (17%)	3 (10%)
Female	25 (83%)	28 (90%)
Race		
Asian/African	5 (17%)	0 (0%)
Coloured	1 (3%)	0 (0%)
Indian	12 (40%)	1 (3%)
White	12 (40%)	30 (97%)
Age range (years)		
56–60	0 (0%)	1 (3%)
61–65	0 (0%)	0 (0%)
66–70	3 (10%)	9 (29%)
71–75	11 (37%)	13 (42%)
76–80	16 (53%)	8 (26%)
Duration of RA (years)		
1–5 years	9 (30%)	3 (10%)
6–10 years	8 (27%)	6 (19%)
11–15 years	1 (3%)	6 (19%)
>16 years	12 (40%)	8 (26%)
Unknown	0 (0%)	8 (26%)

*Source*: Authors’ own work

In Table 2, both groups had a majority of female participants between the ages of 71 and 75 years. There was a preponderance of White and Indian participants in both groups, with equal numbers of Indians (40%) and Whites (40%) in the KT group. The chronicity of the condition was evident in both groups with most participants diagnosed with RA for more than 6 years. More participants in both groups were diagnosed with RA for longer than 16 years.

MCP joints showed a significant decrease in pain on the VAS (*p* = 0.01) and the MHQ (*p* = 0.01) when compared before and after (i.e. week 3 and week 7, respectively) application of Kinesio Taping^®^. In addition, a significant increase in MCP ulnar deviation was found bilaterally (*p* = 0.001 bilaterally) following Kinesio Taping^®^.

Although an increase in grip strength was seen in the mean score (right hand: 8.80–9.43; left hand: 6.77–7.40), this was not statistically significant (right hand: *p* = 0.76; left hand: *p* = 0.14). The Kinesio Taping^®^ group did not show an improvement in the overall score of the MHQ between the assessments completed at week 3 and week 7 (*p* = 0.18).

There was no significant difference in pain in the JP group over time using either the VAS (*p* = 0.20) or the MHQ (*p* = 0.33). No significant difference was noted for MCP ulnar deviation prior to and after the JP intervention. However, joint protection in the JP group was found to significantly improve grip strength in both the right and left hands (*p* = 0.001 bilaterally). In addition, a significant difference improvement was noted in performance in activities of daily living (ADL) (*p* = 0.01) and in work (*p* = 0.01), but the overall MHQ score did not show a significant difference (*p* = 0.24).

The mean scores on the VAS and MHQ for the KT group at week 7 were higher for the outcomes of pain, MCP ulnar deviation (right and left hands), overall MHQ, overall bilateral hand function, overall ADL and satisfaction when compared with the JP group. Cohen’s effect size for these outcomes indicated trivial to small clinical difference between the two interventions. However, Cohen’s effect size values (right hand: *d* = -0.6; left hand: *d* = -1.02) indicated a moderate clinically relevant difference between the two interventions for grip strength. This is also indicated as a significant difference in improvement in grip strength between the means of the JP and Kinesio Taping^®^ groups at week 7 (right hand: *p* = 0.01; left hand: *p* = 0.001). Scores for all other outcomes did not indicate any significant differences.

## Discussion

Various interventions are used in physiotherapy and occupational therapy to decrease inflammation and manage pain to facilitate the release of endogenous opioids in patients diagnosed with RA. Bradley and Adams (2013) state that pain in RA is primarily as a result of inflammation as well as the pathomechanics of RA causing poor support of joints by ligaments, contact between the bones in the joints and osteophyte formation within or around soft tissue. In addition, inflammation in the joint capsules results in stretching of the tissues around the joint, causing further pain (Alter, Feldon & Terrono 2011). Reeve and McArthur (2013) indicate that chronic rheumatic pain of different types (inflammatory, biomechanical and neuropathic) can lead to central sensitisation. This is caused by prolonged inflammation and biomechanical changes, resulting in continuous stimulation of the nociceptors which affects change in the central nervous system.

In this study, Kinesio Taping^®^ has shown a significant decrease in pain at the MCP joints. These findings are similar to those reported previously which found that Kinesio Taping^®^ is effective in providing short-term pain relief to the shoulder, elbow, neck, back, knees and ankles following 4-6 weeks of application (Anandkumar *et al*. 2014; Bae *et al*. 2013; Campolo 2013; Djordjevic *et al*. 2012; Donec & Kriščiūnas 2014; Gonzales-Iglesias 2009; Kalichman, Vered & Volchek 2010; Karatas *et al*. 2012; Kaya, Zinnuroglu & Tugcu 2011; Kuru, Yaliman & Dereli 2012; Paoloni *et al*. 2011; Saavedra-Hernández *et al*. 2012; Simsek *et al*. 2013; Thelen, Dauber & Stoneman 2008). Various theories have been proposed about the mechanism of pain reduction with Kinesio Taping^®^ (Brăteanu 2009; Coopee 2011; Donec & Kriščiūnas 2014; Hancock n.d.; Paoloni *et al*. 2011). It is postulated that stimulation of the mechanoreceptors, thermoreceptors and nociceptors in the skin by the tape causes different responses in the nervous system that may have an effect on pain reduction through the action of endogenous analgesics, spinal inhibition and a decrease in inflammation.

Results from this study indicate that Kinesio Taping^®^ of the MCP joints significantly improved ulnar deviation of the MCP joints bilaterally. There may be two mechanisms responsible for improving the range of movement in the MCP joints. Firstly, the decrease in pain may have led to an improvement in the active range of motion. This effect may be caused by Kinesio Taping^®^ improving mechanical irritation in the soft tissues surrounding the joint, thereby increasing active range of motion. Secondly, it is thought that increased support is given to the ligament structures working on that joint, thereby aligning the joint, reducing pain and improving the range of motion. In this instance, the space correction tape across all the MCP joints may have supported the superficial transverse metacarpal ligament, thereby assisting in aligning the MCP joints. It may be recommended that space correction Kinesio Taping^®^ of the MCP joints be applied in the manner described above to decrease levels of pain in people with RA (i.e. for periods of 3 days at a time, reapplied weekly).

In addition, the reduction in pain may have improved the ulnar deviation of the MCP joint through increasing the degree to which the joint could move within a pain-free range. Despite improvements in ulnar deviation, pain and grip strength, these changes did not facilitate a change in ADL and function. Owing to the chronicity of and adaptation to the condition, it is often difficult for patients to adjust to change (Goodacre & McArthur 2013).

JP programmes in RA are used in order to avoid overuse of the affected joints so as to decrease inflammation and pain and prevent further deformities (Hammond 2013). In prior research, pain, stiffness, function, grip strength and JP behaviour have all been shown to be improved through educational–behavioural JP workshops (Alderson *et al*. 1999; Dures 2012; Hammond 1999, 2013; Hammond, Bryan & Hardy 2008; Hammond & Freeman 2001; Hammond & Lincoln 1999; Iversen *et al*. 2010; Masiero *et al*. 2007). These findings include follow-up research which has ascertained that the results have continued over time, provided that the behaviour change is continued (Hammond *et al*. 2008).

In this study, JP workshops showed a significant improvement in grip strength as well as in the work performance and ADL sections of the MHQ. Grip strength was found to be a significant difference between the two interventions. In addition, a moderate, clinically relevant difference was found in grip strength bilaterally. Grip strength has been correlated with hand function (Adams *et al*. 2010; Vliet-Vlieland *et al*. 1998), and in the JP group, it increased to a mean of 12.65kg. It has been found that grip strength of 20 pounds (9.07 kg) allows individuals to perform most ADLs (Shipham & Pitout 2003) which is possibly the reason why work and ADL scores improved in the MHQ. In addition, it is recommended that practising meaningful activities during the JP workshops can facilitate self-efficacy and understanding of the perceived benefits of using the JP principles (Niedermann *et al*. 2010). Pain, ulnar deviation and the remaining sections of the MHQ did not show significant improvements. One of the reasons for this may be that the participants did not own any of the assistive devices used in the JP workshops and were therefore not implementing and reinforcing ongoing JP principles. Therefore, this ongoing practice of JP could not have a positive impact on pain and ulnar deviation as the behaviour change at home could not occur.

### Limitations of the study

The sample was limited to a small number of participants who were not representative of the population demographics in KwaZulu-Natal. Further, there was no true randomisation of the participants, and the sample was one of convenience; thus, baseline similarities were not assured. The taping was isolated to the MCP joints and no taping to facilitate the forearm extensor muscles was completed. In addition, in this study, the assessment was not conducted within the time period of half an hour to one-and-a-half hours post taping which has previously shown improvement in grip strength. Finally, this study did not incorporate exercises in conjunction with the Kinesio Taping^®^ which had previously been found to improve strength, and this may have affected a possible significant finding in the assessment of grip strength.

## Conclusion

The results of this study demonstrated that Kinesio Taping^®^ of the MCP joints may be an effective modality to improve pain and MCP ulnar deviation in elderly individuals with RA, but these findings require further study in a carefully designed randomised control trial before any conclusions can be drawn on the effectiveness of this modality. It is a convenient conservative modality for individuals with RA as it is relatively cheap and the application can be done by the individuals themselves or the caregiver, if this is carefully demonstrated by a professional. The treatment of a biomedical source of pain through a single intervention does not often ensure a long-term effect, and the focus of therapy continues to be on maximising participation in combination therapies (Reeve & McArthur 2013). It is recommended that Kinesio Taping^®^ could be considered in addition to other conservative interventions, such as JP, assistive devices, splinting, exercise and treatment modalities as intervention for people with RA, once it has been properly tested.
